# Long Term Outcome and Immune Function After Hematopoietic Stem Cell Transplantation for Primary Immunodeficiency

**DOI:** 10.3389/fped.2019.00381

**Published:** 2019-09-24

**Authors:** Andrew R. Gennery, Arjan Lankester, Inborn Errors Working Party (IEWP) of the European Society for Blood and Marrow Transplantation (EBMT)

**Affiliations:** ^1^Institute of Cellular Medicine, Newcastle University, Newcastle upon Tyne, United Kingdom; ^2^Paediatric Stem Cell Transplant Unit, Great North Children's Hospital, Newcastle upon Tyne, United Kingdom; ^3^Stem Cell Transplantation Program, Department of Pediatrics, Willem-Alexander Children's Hospital, Leiden University Medical Center, Leiden, Netherlands

**Keywords:** SCID, thymopoiesis, Artemis, antibody-based conditioning, papillomavirus-associated warts, WAS, CGD

## Abstract

Transplantation techniques for patients with primary immunodeficiencies have improved so that survival from the procedure in many cases is >80%. However, long term complications may arise due to the use or not of conditioning agents. This may result in variable immune reconstitution, the long term effects of chemotherapy, particularly on fertility, and complications relating to the genetic disorder, unresolved by transplantation. For patients with severe combined immunodeficiency (SCID), long term T- and B-lymphocyte immune reconstitution is best achieved after pre-transplant chemotherapy. For patients who receive an unconditioned infusion of donor stem cells, the quality of immune reconstitution depends on the SCID genotype. Long term effects include chemotherapy-induced impaired fertility, and sequelae specific to the genotype. For patients with other primary immunodeficiencies, conditioning is required—sequelae related to direct effects of chemotherapy may be observed. Additional long term effects may be observed due to partial donor chimerism resulting in incomplete eradication of disease, and other geno-specific effects.

## Introduction

Incremental improvements in the approach to transplantation have ensured that survival following allogeneic hematopoietic stem cell transplantation (HSCT) for primary immunodeficiency (PID) is now generally >80% (1–4). More accurate matching at HLA loci using molecular DNA techniques, pre-emptive treatment of viremia, adoption of less toxic condition regimens and pharmacokinetic monitoring (5–8), development of more effective T-lymphocyte depletion methods (9, 10), and more effective treatment of transplant-related complications (11–13) now mean that the majority of patients undergoing HSCT for PID can expect to survive the procedure. As a consequence of this increase survival rate, more emphasis is being put on the quality of long term outcome. An overview on both specific immuno-hematological as well as overall quality of health perspectives is discussed below.

## Severe Combined Immunodeficiency

### Long Term Immune Function

The issue regarding long-term outcome is probably most complex but also best studied in patients with severe combined immunodeficiency (SCID). Severe combined immunodeficiencies are a heterogeneous group of inherited primary immunodeficiencies characterized by absent thymopoiesis due to lack of T-lymphocyte progenitors available to enter or to develop within the thymus, failure of T-lymphocyte maturation and function, and thus severally impaired cellular and humoral acquired immunity. Depending on the genetic defect, recipient B-lymphocytes, and/or Natural Killer cells may be present. The lack of T-lymphocytes, often accompanied by significant infection, and end-organ damage resulted in many patients receiving a stem cell infusion without preparative chemotherapy to empty osteo-medullary and thymic niches. Immune-reconstitution was reported to be variable, and it is now clear that the outcome depends on the specific SCID genotype and thereby the stage in which thymopoiesis is arrested. Mature donor stem cell-derived T-lymphocytes, transferred in the inoculum, populate the periphery, and expand to confer medium-term but finite immune protection, with a restricted T-lymphocyte receptor repertoire. Effective long-term thymopoiesis with T-lymphocyte immune reconstitution requires donor stem cell-derived T-lymphocyte progenitors to enter the thymus and undergo proliferation during the thymocyte double negative (DN) 1 and 2 stages, before DN3, when thymocytes undergo rearrangement of the T-lymphocyte receptor. The lack of recipient T-lymphocytes to facilitate rejection and the concept of host osteo-medullary and thymic environments which are either permissive or non-permissive to engraftment following infusion (14, 15) long provoked debate over approach to treatment. The debate centers around whether infusion of donor stem cells, without administering immunosuppressive and myeloablative pre- transplant chemotherapy conditioning, is equally effective and less toxic/more safe in realizing a long-term cure than transplantation of donor stem cells following administration of myeloablative chemotherapy conditioning. With careful analysis of immune reconstitution in single (16–21), and multi-center cohorts (1, 22, 23), there is emerging clarification about which molecular diagnoses permit adequate long-term thymopoiesis after graft infusion without conditioning, and which genotypes of SCID likely require conditioning for best long-term results.

Infusion without chemo-conditioning of replete marrow from HLA-matched sibling or matched unrelated donors or T-lymphocyte-depleted haplo-identical stem cells from parental donors facilitates thymopoiesis and T-lymphocyte immune reconstitution. This is particularly the case in patients with IL-2 receptor gamma chain (IL-2Rγ)-, janus-associated kinase 3 (JAK3)-, or adenosine deaminase-(ADA) deficient SCID, and leads to good survival (1, 15, 20, 21, 24, 25). Pre-thymic and early intrathymic stromal niches are vacant with no competition between donor progenitors and endogenous, immature, DN thymocytes, which are absent in these SCID conditions. Thymic niches are thus available for donor T-lymphocyte precursor engraftment, leading to sustained donor-derived thymopoiesis in the absence of myelopoiesis (26), which nevertheless may be of finite durability (21). Donor B-lymphocyte engraftment usually fails to occur because the osteo-medullary niche is occupied by recipient B lineage pre-cursors and mature cells. In IL-2Rγ- and JAK3-deficient SCID, recipient B-lymphocytes are functionally impaired (27), and infused patients usually require long-term immunoglobulin replacement. IL-7Rα-deficient patients usually develop B-lymphocyte function, as recipient B-lymphocytes have intact intrinsic function, and produce immunoglobulin in the presence of functioning donor T-lymphocytes. Patients with ADA-deficient SCID often develop cellular and humoral immune reconstitution in the absence of conditioning, possibly because the local toxic effects of ADA deficiency on the marrow act as “auto-conditioning” combined with a selective growth advantage and permit donor stem cell engraftment in the absence of chemotherapy. Rare patients with a CID-phenotype due to ADA deficiency require chemo-conditioning to achieve engraftment.

Thymopoiesis and humoral immunity is infrequently observed in unconditioned patients with NK+ SCID due to defects in recombination activating genes 1 and 2- (*RAG1/2*) and *DCLRE1C* (Artemis). In these patients, early T-lymphocyte development is arrested at later stages of committed T-lymphocyte differentiation creating a non-permissive thymic environment occupied with high numbers of DN2/DN3 cells competing with donor T-lymphocyte progenitors for thymic niches. As a consequence SCID patients with these genetic defects are refractory to thymic and B lineage reconstitution following transplantation without conditioning because of competition for thymic and osteo-medullary niches. Following infusion of donor cells without conditioning, immune reconstitution is poor; T-lymphocyte engraftment relies on post-thymic peripheral T-lymphocyte expansion, with a failure of thymopoiesis, and a restricted T-lymphocyte receptor repertoire (17, 18, 28).

Together, in the long-term, in unconditioned transplants, superior thymopoiesis is observed in IL-2Rγ-, JAK3-, IL-7 receptor alpha- (IL-7Rα), or ADA- deficient SCID compared with recombination activating genes 1 and 2- (*RAG1/2*) and *DCLRE1C* (Artemis) -deficient SCID. However, in the absence of pre-conditioning chemotherapy, donor B-lymphocyte, and myeloid chimerism is generally absent in either group (14), with the exception of ADA- deficient SCID (24).

As there is no survival difference between those patients receiving chemotherapy conditioning and those receiving either immunosuppression only, or no preparative regimen (1, 23), at present, a chemotherapy conditioning preparation is preferred to achieve donor stem cell engraftment and thus durable immune reconstitution if the physical condition of the patient will allow this. Durable T-lymphocyte reconstitution is associated with better survival (29), good T-lymphocyte reconstitution at 1 to 2 years post-HSCT is associated with better T-lymphocyte long-term immune reconstitution (18), and high T-lymphocyte receptor excision (TREC) circle counts, markers of thymopoiesis, at 6 months associate with robust long-term T-lymphocyte reconstitution (1, 16, 17, 20, 21).

The advantageous effect of conditioning on long term graft and immune function also raises new challenges in the context of the observation that infants treated in the 1st months of life, before the onset of infection, have the most favorable outcome, with a survival of >90%, and fewer long-term infectious, and immune-related complications (22, 30). This is particularly relevant given the ongoing implementation of SCID newborn screening (NBS) programs in many countries worldwide (*refer to chapters on SCID/NBS*). Whilst there is unease in the transplant community about administering chemotherapy to young infants to optimize engraftment and reconstitution, it is not clear what the long term effects of such administration are, or at what age these effects are likely to be less significant. One study using treosulfan and fludarabine in infants <5 months of age showed good survival and no significant early toxicity, although long term effects will need to be evaluated (6). The routine adoption of pharmacokinetic studies during chemotherapy delivery may help answer some of the questions.

Although we may now have a better understanding of how to avoid poor graft function, there are many surviving patients living with the effects of poor immunity or sequelae of post-transplant complications, such as graft-vs. -host disease. There are few published data on this—one single center cohort study of 90 patients describes chronic graft-vs. -host disease in 10% of patients (31). Additionally, patients were described with autoimmune and inflammatory conditions, and significant infections. Within this cohort, about one third of patients did require any form of long-term treatment (31). In another large cohort study of 124 patients, over half of whom had IL-2Rγ- and JAK3-deficient SCID, 86% of 111 survivors with follow up were considered healthy by the physicians, although 53% required long-term immunoglobulin replacement indicative for incomplete immune correction. Other long-term complications reported included autoimmunity, hypothyroidism, malignancy, and developmental delay (30).

### Long Term Sequelae in SCID Patients

In transplanted SCID patients several long-term sequelae are directly related to the underlying genetic defect as part of their syndromal disease, and occur independent of the immune-hematological defect, and reconstitution characteristics after stem cell therapy. Patients with radiosensitive SCID due to mutations in *DCLRE1C* experience significant long-term sequelae including growth, endocrine and dental abnormalities, pancreatic insufficiency, pulmonary fibrosis, as well as increased mortality, if conditioning regimens contain alkylating agents (1, 32). On the other hand, unconditioned transplants result in poor immune reconstitution (*vide supra*), and the best approach to treating these patients has yet to be determined. For Artemis deficient SCID, but also similar vulnerable patients, innovative targeted therapy directed toward the stem cell niches on marrow, and thymus using antibody-based conditioning (33–35) may achieve some myeloid engraftment without the chemo-conditioning associated toxicities.

Patients with ADA-deficiency, a systemic metabolic disease in which HSCT only corrects the immune defect, cognitive, behavioral, or other neurological outcomes appear not to be affected by transplantation, although it is not possible to know if there is some amelioration (36–38). Similarly, SCID variants caused by DNA ligase IV and Cernunnos are associated with an intrinsic defect associated with long term neurological impairment which will not be corrected by HSCT. Many patients with reticular dysgenesis have an associated sensorineural deafness, related to the defect in adenylate kinase 2, which is not corrected by HSCT but may be ameliorated by cochlear implants (39, 40).

Patients with IL-2Rγ- and JAK3-deficient SCID are at risk of developing extensive cutaneous human papillomavirus-associated warts which are recalcitrant to treatment, and not clearly associated with degree of donor chimerism and the level of T-lymphocyte reconstitution (41, 42) but also seen with low numbers of NK cells. Although the pathophysiologic mechanism is not completely understood, evidence has been provided that lack of the common γ chain function in non-hematologic cells, i.c., keratinocytes may impair the secretion of chemokines that may guide the influx of protective immune cells. A canine model of IL-2Rγ-deficient SCID demonstrates a similar problem, in which malignant transformation has been demonstrated (43)—patients will require careful long-term follow up.

### Non-SCID Immunodeficiencies

Data on long-term follow up and immune function in non-SCID primary immunodeficiencies are more scarce than for SCID. Non-SCID patients usually require full or reduced intensity chemotherapy conditioning to achieve donor stem cell engraftment and myeloid chimerism for cure of their immune disorder. In contrast to SCID where mixed or split chimerism as a result of the selective growth advantage of donor T- and B lymphocytes may already be curative this does not necessarily apply for non-SCID immunodeficiencies. In a number of these disease categories described hereafter, mixed donor/recipient stem cell chimerism may result in only partial correction of the immune disorder and thus occurrence or persistence of concomitant long term complications.

The possibly unfavorable impact of mixed chimerism on correction of the immune disorder has been reported in Wiskott Aldrich syndrome, a syndromal combined immune deficiency due to defects in the *WASP* gene which plays a pivotal role in actin cytoskeleton. There is no selective advantage to cells harboring the wild type gene, and so partial donor stem cell chimerism results in a mixed recipient/donor population of antigen presenting cells, and lymphocytes. The presence of mixed donor and recipient chimerism has been reported to be associated with late onset autoimmunity in these patients (44, 45). Although the exact pathophysiologic mechanism for this autoimmunity remains to be defined it seems best to aim to achieve high-level or complete donor chimerism.

In the X-linked form of chronic granulomatous disease recent data suggest that, because of random lyonisation, female carriers of X-linked chronic granulomatous disease are at significant risk of experiencing autoimmunity, fatigue, anxiety and depression, likely because of inflammation caused by the genetically faulty phagocytes (46–48). Neutrophil function of <10% was highly associated with an increased risk of infection (47). Although to date, there are no reported similar symptoms in transplanted patients with mixed donor chimerism, nevertheless, these findings suggest that high or complete donor chimerism is desirable, and ideally X-linked disease carriers are not used as donors if other suitable donors are available.

A growing group of primary immune disorders that may benefit from allogeneic stem cell therapy and may be even gene therapy is represented by patients with monogenic diseases associated with autoimmunity and inflammatory symptoms. Whilst the numbers of patients reported are few, data are emerging to suggest that residual dysfunctional host immunity in these immune dysregulation diseases may cause persistent autoimmunity and inflammation and therefore high or complete donor chimerism may be required to abolish disease symptoms, especially in those diseases where the genetic defect results on an activated (gain-of-function) instead of a non-functional gene product. Diseases thus described include Activated PI3K Delta Syndrome (49), CTLA4 deficiency (50), LRBA deficiency (51), and STAT1 gain-of-function disease (52). The degree of donor chimerism required to cure these different disease entities is yet to be determined. However, based on the pathophysiologic mechanism of some of these diseases, full donor chimerism may be required to avoid disease manifestations due to residual recipient immunity.

### Complications in SCID and Non-SCID

#### Infertility and Other Endocrine Complications

Similar as for many other HSCT indications requiring chemotherapy-based conditioning, a significant concern is that of fertility. There are few data regarding this issue specifically in patients transplanted for PID, partly because a long follow up is required before the reproductive capability of patients transplanted as young children is known, should the patient even wish to have children. Furthermore, conditioning regimens evolve over time, and the fertility of patients now under investigation often reflects historic conditioning regimens. Finally, there may be differences in fertility outcomes depending on whether chemotherapy was given to a young infant or an adolescent. In one study of patients, including 68 patients transplanted for non-malignant disease, spontaneous puberty was achieved in all males, and 90% of females who received a reduced intensity conditioning regimen containing fludarabine and melphalan, compared with 56% of females, and 89% of males who received a myeloablative regimen containing busulfan, and cyclophosphamide (53). In a recent study documenting transplant outcomes of 55 children and adolescents undergoing stem cell transplantation for chronic granulomatous disease from a single center, 6 of 11 survivors older than 21 years at the time of the study had experienced successful unassisted pregnancy themselves or with their partner, of whom 4 had received busulfan and cyclophosphamide, 1 received busulfan and fludarabine, and 1 received fludarabine and melphalan (54). Two adults transplanted for chronic granulomatous disease were reported as fathering children after receiving a targeted dose regimen of busulfan with fludarabine (5). A recent study of the European Society for Blood and Marrow Transplantation suggests that gonadal damage may be less following treosulfan-based conditioning compared to busulfan-based conditioning, although the number of patients receiving treosulfan was much smaller (55). Although these data are few, they suggest that infertility is not inevitable following conditioned HSCT for PID, and that the outlook may be considerably better than previously feared. There may be differences between conditioning regimens, and careful long term follow up should be continued.

Thyroid dysfunction may occur as an immune-mediated manifestation of residual primary disease or due to chemotherapy related toxicity after HSCT and not related to the underlying immune disorder (56).

#### Secondary Malignancies

A survey by the European Society for Blood and Marrow Transplantation has estimated the incidence of myelodysplastic syndrome or acute myeloid leukemia to be 1.2:1,000 in transplants for malignancy, mostly occurring within 4 years of HSCT (57). However, these patients present with malignancy, usually receive more intense chemotherapy than that administered as preparative pre-conditioning, and often have irradiation as part of their treatment. The risk of a non-post-transplant lymphoproliferative malignancy post-transplantation in patients who were transplanted for PID is less well-understood. The presence of a PID is associated with an increased risk of malignancy, around 4–5 times greater than in age-matched controls (58). Risks of developing a malignancy post-transplantation may be related to the underlying genetic disease, the tissue distribution of genetic defect (confined to the hematopoietic compartment or more wide-spread), previous graft vs. host disease, viral infections, and the extent of donor chimerism, and quality of restored immune function. Three studies together found 21 of 3,340 (0.6%) patients developed a malignancy (59–61). Interestingly, in a large series of patients transplanted for systemic DNA double strand-breakage repair disorders, which predispose to malignancy, there were no reported cases of secondary tumors, although median follow up was only 35 months (62). Collectively, these data demonstrate that there is a small but real risk of malignancy developing in this post-transplant cohort and re-enforce the need for careful long term surveillance. This will be particularly important for patients in whom malignancy risk is a significant part of the immunodeficiency, such as those patients with systemic DNA double strand-breakage repair disorders, or cartilage hair hypoplasia.

#### Quality of Life

For many years, studies of patients receiving hematopoietic stem cell transplantation for PID have concentrated on survival, and more recently on the quality of immune reconstitution. Long-term quality of life is an important consideration for patients transplanted for SCID. There are few studies examining this, and whilst one found a diminished quality of life compared to normal controls (38), two others found that quality of life was related to requirement of on-going treatment, particularly immunoglobulin replacement, with those patients who were on no medication reporting a normal life quality (20, 21). Both in SCID and non-SCID patients, patient reported outcome on quality of life is an important measure of treatment outcome, largely ignored in this field. There are no studies comparing quality of life before and after transplantation, and only one that compares transplanted, and non-transplanted age-matched pediatric cohorts in chronic granulomatous disease (63). This study showed a normal quality of life in patients who had been transplanted, compared with those receiving conventional therapy. In the last decade HSCT has become an increasingly safe therapeutic modality. In addition to the traditional severe immune deficiency syndromes in which survival is the primary goal, HSCT is more often considered in the broadening spectrum of patients with severe/profound but not-acute life threatening primary immune disorders. In these chronic disabling diseases HSCT primarily aims to improve quality of life and add value both from the patient and physician perspective. More studies performed in close collaboration between physicians and patients are required in the growing field of these rare primary immune disorders to measure the impact of stem cell transplantation and alterative (e.g., biologicals/GT) on long term disease control as well as quality of life.

## Conclusion

There is no doubt that hematopoietic stem cell transplantation has saved and transformed the lives of many patients with PID. However, although many patients are able to subsequently live normal lives, significant post-transplant sequelae reduce life expectancy, or impair the quality of life in a subgroup of patients. Given that many patients have extremely rare immunodeficiencies, the number of patients in follow up is small, with a short time period of post-transplant follow up. Continued careful observation and enhanced surveillance is required to learn more about the effects of our transplant techniques on these patients, and to improve long term outcomes.

## Author Contributions

AG and AL contributed to the inception and design of the review and wrote and edited it.

### Conflict of Interest

The authors declare that the research was conducted in the absence of any commercial or financial relationships that could be construed as a potential conflict of interest.

## References

[1] Haddad E Logan BR Griffith LM Buckley RH Parrott RE Prockop SE SCID genotype and 6-month posttransplant CD4 count predict survival and immune recovery: a PIDTC retrospective study. Blood. (2018) 132:1737–49. 10.1182/blood-2018-03-840702 30154114 PMC6202916

[2] Ferrua F Galimberti S Courteille V Slatter MA Booth C Moshous D on behalf of SCETIDE, PIDTC, EBMT & ESID IEWP. Hematopoietic stem cell transplantation for CD40 ligand deficiency: results from an EBMT/ESID-IEWP-SCETIDE-PIDTC Study. J Allergy Clin Immunol. (2019) 143:2238–53. 10.1016/j.jaci.2018.12.1010 30660643

[3] Cole T Pearce MS Cant AJ Cale CM Goldblatt D Gennery AR Clinical outcome in children with chronic granulomatous disease managed conservatively or with hematopoietic stem cell transplantation. J Allergy Clin Immunol. (2013) 132:1150–5. 10.1016/j.jaci.2013.05.031 23870668

[4] Marsh RA Hebert KM Keesler D Boelens JJ Dvorak CC Eckrich MJ. Practice pattern changes and improvements in hematopoietic cell transplantation for primary immunodeficiencies. J Allergy Clin Immunol. (2018) 142:2004–7. 10.1016/j.jaci.2018.08.010 30170121 PMC6289686

[5] Güngör T Teira P Slatter M Stussi G Stepensky P Moshous D Reduced-intensity conditioning and HLA-matched haemopoietic stem-cell transplantation in patients with chronic granulomatous disease: a prospective multicentre study. Lancet. (2014) 838:436–48. 10.1016/S0140-6736(13)62069-3 24161820

[6] Slatter M Rao K Abd Hamid IJ Nademi Z Chiesa R Elfeky R Treosulfan and fludarabine conditioning for haematopoietic stem cell transplantation in children with Primary Immunodeficiency: UK experience. Biol Blood Marrow Transplant. (2018) 24:529–36. 10.1016/j.bbmt.2017.11.009 29155317

[7] van der Stoep MYEC Bertaina A Ten Brink MH Bredius RG Smiers FJ Wanders DCM. High interpatient variability of treosulfan exposure is associated with early toxicity in paediatric HSCT: a prospective multicentre study. Br J Haematol. (2017) 179:772–80. 10.1111/bjh.14960 29048102

[8] Admiraal R van Kesteren C Jol-van der Zijde CM Lankester AC Bierings MB Egberts TC. Association between anti-thymocyte globulin exposure and CD4+ immune reconstitution in paediatric haemopoietic cell transplantation: a multicentre, retrospective pharmacodynamic cohort analysis. Lancet Haematol. (2015) 2:e194–203. 10.1016/S2352-3026(15)00045-9 26688094

[9] Balashov D Shcherbina A Maschan M Trakhtman P Skvortsova Y Shelikhova L. Single-center experience of unrelated and haploidentical stem cell transplantation with TCRα*β* and CD19 depletion in children with primary immunodeficiency syndromes. Biol Blood Marrow Transplant. (2015) 21:1955–62. 10.1016/j.bbmt.2015.07.008 26187864

[10] Shah R Elfeky R Nademi Z Qasim W Amrolia P Chiesa R HLA-haploidentical and mismatched unrelated donor hematopoietic stem cell transplantation after TCRα*β*^+^/CD19^+^ cell depletion in children with primary immune deficiencies: the UK experience. J Allergy Clin Immunol. (2018) 141:1417–26.e1. 10.1016/j.jaci.2017.07.008 28780238

[11] Kernan NA Grupp S Smith AR Arai S Triplett B Antin JH. Final results from a defibrotide treatment-IND study for patients with hepatic veno-occlusive disease/sinusoidal obstruction syndrome. Br J Haematol. (2018) 181:816–27. 10.1111/bjh.15267 29767845 PMC6032999

[12] Das-Gupta E Dignan F Shaw B Raj K Malladi R Gennery A. Extracorporeal photopheresis for treatment of adults and children with acute GVHD: UK consensus statement and review of published literature. Bone Marrow Transplant. (2014) 49:1251–8. 10.1038/bmt.2014.106 24887389

[13] Ball LM Bernardo ME Roelofs H van Tol MJ Contoli B Zwaginga JJ. Multiple infusions of mesenchymal stromal cells induce sustained remission in children with steroid-refractory, grade III-IV acute graft-versus-host disease. Br J Haematol. (2013) 163:501–9. 10.1111/bjh.12545 23992039

[14] Hassan A Lee P Maggina P Xu JH Moreira D Slatter M. Host natural killer immunity is a key indicator of permissiveness for donor cell engraftment in patients with severe combined immunodeficiency. J Allergy Clin Immunol. (2014) 133:1660–6. 10.1016/j.jaci.2014.02.042 24794685 PMC4048544

[15] Dvorak CC Hassan A Slatter MA Hönig M Lankester AC Buckley RH. Comparison of outcomes of hematopoietic stem cell transplantation without chemotherapy conditioning by using matched sibling and unrelated donors for treatment of severe combined immunodeficiency. J Allergy Clin Immunol. (2014) 134:935–43.e15. 10.1016/j.jaci.2014.06.021 25109802 PMC4186906

[16] Sarzotti-Kelsoe M Win CM Parrott RE Cooney M Moser BK Roberts JL. Thymic output, T cell diversity and T cell function in long-term human SCID chimeras. Blood. (2009) 114:1445–53. 10.1182/blood-2009-01-199323 19433858 PMC2727406

[17] Borghans JA Bredius RG Hazenberg MD Roelofs H Jol-van der Zijde EC Heidt J. Early determinants of long-term T-cell reconstitution after hematopoietic stem cell transplantation for severe combined immunodeficiency. Blood. (2006) 108:763–9. 10.1182/blood-2006-01-009241 16822903

[18] Cavazzana-Calvo M Carlier F Le Deist F Morillon E Taupin P Gautier D. Long-term T-cell reconstitution after hematopoietic stem-cell transplantation in primary T-cell-immunodeficient patients is associated with myeloid chimerism and possibly the primary disease phenotype. Blood. (2007) 109:4575–81. 10.1182/blood-2006-07-029090 17272510

[19] Mazzolari E Forino C Guerci S Imberti L Lanfranchi A Porta F. Long-term immune reconstitution and clinical outcome after stem cell transplantation for severe T-cell immunodeficiency. J Allergy Clin Immunol. (2007) 120:892–9. 10.1016/j.jaci.2007.08.007 17825895

[20] Abd Hamid IJ Slatter MA McKendrick F Pearce MS Gennery AR Long-term outcome IL2RG/JAK3 SCID post-hematopoietic stem cell transplantation: a cohort report. Blood. (2017) 129:2198–201. 10.1182/blood-2016-11-748616 28209722

[21] Abd Hamid IJ Slatter MA McKendrick F Pearce MS Gennery AR Long term health outcome and quality of life post-HSCT for IL7Rα-, Artemis-, RAG1- and RAG2-deficient severe combined immunodeficiency: a single centre report. J Clin Immunol. (2018) 38:727–32. 10.1007/s10875-018-0540-9 30105620

[22] Pai S-Y Logan BR Griffith LM Buckley RH Parrott RE Dvorak CC. Transplantation outcomes for severe combined immunodeficiency, 2000–2009. N Engl J Med. (2014) 371:434–46. 10.1056/NEJMoa1401177 25075835 PMC4183064

[23] Gennery AR Slatter MA Grandin L Taupin P Cant AJ Veys P. Transplantation of hematopoietic stem cells and long-term survival for primary immunodeficiencies in Europe: entering a new century, do we do better? J Allergy Clin Immunol. (2010) 126:602–10. 10.1016/j.jaci.2010.06.015 20673987

[24] Hassan A Booth C Brightwell A Allwood Z Veys P Rao K. Outcome of hematopoietic stem cell transplantation for adenosine deaminase-deficient severe combined immunodeficiency. Blood. (2012) 120: 3615–24 10.1182/blood-2011-12-396879 22791287

[25] Buckley RH Schiff SE Schiff RI Markert L Williams LW Roberts JL. Hematopoietic stem-cell transplantation for the treatment of severe combined immunodeficiency. N Engl J Med. (1999) 340:508–16 10.1056/NEJM199902183400703 10021471

[26] Prockop SE Petrie HT. Regulation of thymus size by competition for stromal niches among early T cell progenitors. J Immunol. (2004) 173:1604–11. 10.4049/jimmunol.173.3.1604 15265888

[27] Recher M Berglund LJ Avery DT Cowan MJ Gennery AR Smart J. IL-21 is the primary common gamma chain-binding cytokine required for human B-cell differentiation *in vivo*. Blood. (2011) 118:6824–35. 10.1182/blood-2011-06-362533 22039266 PMC3338166

[28] Slatter MA Brigham K Dickinson AM Harvey HL Barge D Jackson A Long-term immune reconstitution following anti- CD52-treated or anti-CD34-treated haematopoietic stem cell transplant for severe T lymphocyte immunodeficiency. J Allergy Clin Immunol. (2008) 121:361–7. 10.1016/j.jaci.2007.10.035 18086494

[29] Haddad E Landais P Friedrich W Gerritsen B Cavazzana-Calvo M Morgan G. Long- term immune reconstitution and outcome after HLA-nonidentical T-cell-depleted bone marrow transplantation for severe combined immunodeficiency: a European retrospective study of 116 patients. Blood. (1998) 91:3646–53. 9573000

[30] Railey MD Lokhnygina Y Buckley RH Long-term clinical outcome of patients with severe combined immunodeficiency who received related donor bone marrow transplants without pretransplant chemotherapy or post-transplant GVHD prophylaxis. J Pediatr. (2009) 155:834–40. 10.1016/j.jpeds.2009.07.049 19818451 PMC2784223

[31] Neven B Leroy S Decaluwe H Le Deist F Picard C Moshous D. Long-term outcome after hematopoietic stem cell transplantation of a single-center cohort of 90 patients with severe combined immunodeficiency. Blood. (2009) 113:4114–24. 10.1182/blood-2008-09-177923 19168787

[32] Schuetz C Neven B Dvorak CC Leroy S Ege MJ Pannicke U. SCID patients with ARTEMIS vs RAG deficiencies following HCT: increased risk of late toxicity in ARTEMIS-deficient SCID. Blood. (2014) 123:281–9. 10.1182/blood-2013-01-476432 24144642 PMC3953035

[33] Schulz AS Glatting G Hoenig M Schuetz C Gatz SA Grewendorf S. Radioimmunotherapy-based conditioning for hematopoietic cell transplantation in children with malignant and nonmalignant diseases. Blood. (2011) 117:4642–50. 10.1182/blood-2010-06-284349 21325170

[34] Straathof KC Rao K Eyrich M Hale G Bird P Berrie E. Haemopoietic stem-cell transplantation with antibody-based minimal-intensity conditioning: a phase 1/2 study. Lancet. (2009) 374:912–20. 10.1016/S0140-6736(09)60945-4 19729196

[35] Derderian SC Togarrati PP King C Moradi PW Reynaud D Czechowicz A. *In utero* depletion of fetal hematopoietic stem cells improves engraftment after neonatal transplantation in mice. Blood. (2014) 124:973–80. 10.1182/blood-2014-02-550327 24879814 PMC4126335

[36] Hönig M Albert MH Schulz A Sparber-Sauer M Schütz C Belohradsky B. Patients with adenosine deaminase deficiency surviving after hematopoietic stem cell transplantation are at high risk of CNS complications. Blood. (2007) 109:3595–602. 10.1182/blood-2006-07-034678 17185467

[37] Rogers MH Lwin R Fairbanks L Gerritsen B Gaspar HB. Cognitive and behavioral abnormalities in adenosine deaminase deficient severe combined immunodeficiency. J Pediatr. (2001) 139:44–50. 10.1067/mpd.2001.115023 11445793

[38] Titman P Pink E Skucek E O'Hanlon K Cole TJ Gaspar J. Cognitive and behavioral abnormalities in children after hematopoietic stem cell transplantation for severe congenital immunodeficiencies. Blood. (2008) 112:3907–13. 10.1182/blood-2008-04-151332 18645040

[39] Lagresle-Peyrou C Six EM Picard C Rieux-Laucat F Michel V Ditadi A. Human adenylate kinase 2 deficiency causes a profound hematopoietic defect associated with sensorineural deafness. Nat Genet. (2009) 41:106–11. 10.1038/ng.278 19043416 PMC2612090

[40] Pannicke U Hönig M Hess I Friesen C Holzmann K Rump EM. Reticular dysgenesis (aleukocytosis) is caused by mutations in the gene encoding mitochondrial adenylate kinase 2. Nat Genet. (2009) 41:101–5. 10.1038/ng.265 19043417

[41] Laffort C Le Deist F Favre M Caillat-Zucman S Radford-Weiss I Debré M Severe cutaneous papillomavirus disease after haemopoietic stem-cell transplantation in patients with severe combined immune deficiency caused by common gamma cytokine receptor subunit or JAK-3 deficiency. Lancet. (2004) 363:2051–4. 10.1016/S0140-6736(04)16457-X 15207958

[42] Kamili QUA Seeborg FO Saxena K Nicholas SK Banerjee PP Angelo LS. Severe cutaneous human papillomavirus infection associated with natural killer cell deficiency following stem cell transplantation for severe combined immunodeficiency. J Allergy Clin Immunol. (2014) 134:1451–3.e1. 10.1016/j.jaci.2014.07.009 25159470 PMC5182041

[43] Goldschmidt MH Kennedy JS Kennedy DR Yuan H Holt DE Casal ML. Severe papillomavirus infection progressing to metastatic squamous cell carcinoma in bone marrow-transplanted X-linked SCID dogs. J Virol. (2006) 80:6621–8. 10.1128/JVI.02571-05 16775349 PMC1488951

[44] Ozsahin H Cavazzana-Calvo M Notarangelo LD Schulz A Thrasher AJ Mazzolari E. Long-term outcome following hematopoietic stem-cell transplantation in Wiskott-Aldrich syndrome: collaborative study of the European society for immunodeficiencies and European Group for blood and marrow transplantation. Blood. (2008) 111:439–45. 10.1182/blood-2007-03-076679 17901250

[45] Moratto D Giliani S Bonfim C Mazzolari E Fischer A Ochs HD. Long-term outcome and lineage-specific chimerism in 194 patients with Wiskott-Aldrich syndrome treated by hematopoietic cell transplantation in the period 1980–2009: an international collaborative study. Blood. (2011) 118:1675–84. 10.1182/blood-2010-11-319376 21659547 PMC3156052

[46] Battersby AC Braggins H Pearce MS Cale CM Burns SO Hackett S Infective, inflammatory and autoimmune manifestations of disease in X-linked carriers of chronic granulomatous disease in the United Kingdom. J Allergy Clin Immunol. (2017) 140:628–30.e6. 10.1016/j.jaci.2017.02.029 28343844

[47] Marciano BE Zerbe CS Falcone EL Ding L DeRavin SS Daub J. X-linked carriers of chronic granulomatous disease: illness, lyonization and stability. J Allergy Clin Immunol. (2018) 141:365–71. 10.1016/j.jaci.2017.04.035 28528201

[48] Battersby AC Braggin H Pearce MS McKendrick F Campbell M Burns S. Health-related quality of life and emotional health in X-linked carriers of chronic granulomatous disease in the United Kingdom. J Clin Immunol. (2019) 39:195–9. 10.1007/s10875-019-00607-6 30868346 PMC6445821

[49] Nademi Z Slatter MA Dvorak CC Neven B Fischer A Suarez F on behalf of the inborn error working party of EBMT and ESID haematopoietic stem cell transplant in patients with activated PI3K delta syndrome. J Allergy Clin Immunol. (2017) 139:1046–9. 10.1016/j.jaci.2016.09.040 27847301

[50] Slatter MA Engelhardt KR Burroughs LM Arkwright PD Nademi Z Skoda-Smith S Haematopoietic stem cell transplantation for CTLA-4 deficiency. J Allergy Clin Immunol. (2016) 138:615–9.e1. 10.1016/j.jaci.2016.01.045 27102614

[51] Seidel MG Böhm K Dogu E Worth A Thrasher A Florkin B on behalf of the inborn errors working party of the european group for blood and marrow transplantation treatment of severe forms of LPS-responsive beige-like anchor protein (LRBA) deficiency by allogeneic hematopoietic stem cell transplantation. J Allergy Clin Immunol. (2018) 141:770–5.e1. 10.1016/j.jaci.2017.04.023 28502825

[52] Leiding JW Okada S Hagin D Abinun M Shcherbina A Balashov DN on behalf of IEWP of EBMT and PIDTC Hematopoietic stem cell transplantation in patients with gain of function STAT1 mutation. J Allergy Clin Immunol. (2018) 141:704–17.e5. 10.1016/j.jaci.2017.03.049 28601685 PMC7802430

[53] Panasuik A Nussey S Veys P Amrolia P Rao K Krawczuk-Rybak M Gonadal function and fertility after stem cell transplantation in childhood: comparison of a reduced intensity conditioning regimen containing melphalan with a myeloablative regimen containing busulfan. Br J Haematol. (2015) 170:719–26. 10.1111/bjh.13497 25974284

[54] Lum SH Flood T Hambleton S McNaughton P Watson H Abinun M Two decades of excellent transplant survival in children with chronic granulomatous disease: a report from a supraregional immunology transplant center in Europe. Blood. (2019) 133:2546–9. 10.1182/blood.2019000021 30952673

[55] Faraci M Diesch T Labopin M Dalissier A Lankester A Gennery A Gonadal function after Busulphan compared to Treosulphan in children and adolescents undergoing allogeneic haematopoietic stem cell transplantation. On Behalf of Pediatric and Transplant-Related Complications and Quality of Life EBMT Working Parties. Biol Blood Marrow Transplant. (2019). 10.1016/j.bbmt.2019.05.005 31082473

[56] Slatter MA Gennery AR Cheetham TD Bhattacharya A Crooks BN Flood TJ. Thyroid dysfunction after bone marrow transplantation for primary immunodeficiency without the use of total body irradiation in conditioning. Bone Marrow Transplant. (2004) 33:949–53. 10.1038/sj.bmt.1704456 15004542

[57] Hertenstein B Hambach L Bacigalupo A Schmitz N McCann S Slavin S. Development of leukemia in donor cells after allogeneic stem cell transplantation—a survey of the European group for blood and marrow transplantation (EBMT). Haematologica. (2005) 90:969–75. 15996934

[58] Bomken S van der Werff Ten Bosch J Attarbaschi A Bacon CM Borkhardt A Boztug K Current understanding and future research priorities in malignancy associated with primary immunodeficiency and DNA repair disorders: the perspective of an interdisciplinary working group. Front Immunol. (2018) 9:2192 10.3389/fimmu.2018.02912 30619276 PMC6299915

[59] Kamani NR Kumar S Hassebroek A Eapen M LeRademacher J Casper J. Malignancies after hematopoietic cell transplantation for primary immune deficiencies: a report from the center for international blood and marrow transplant research. Biol Blood Marrow Transplant. (2011) 17:1783–9. 10.1016/j.bbmt.2011.05.008 21658461 PMC3370408

[60] Nelson AS Vadjic CM Ashton LJ Le Marsney RE Nivison-Smith I Wilcox L. Incident cancers and late mortality in Australian children treated by allogeneic stem cell transplantation for non-malignant diseases. Pediatr Blood Cancer. (2017) 64:197–202. 10.1002/pbc.26219 27671369

[61] Unni MNM Elfeky R Rao K Nademi Z Chiesa R Amrolia P Non-PTLD malignancy post HSCT in patients with primary immunodeficiency: UK experience. J Allergy Clin Immunol. (2018) 141:2319–21e.1. 10.1016/j.jaci.2018.02.038 29524534

[62] Slack J Albert MH Balashov D Belohradsky BH Bertaina A Bleesing J Outcome of haematopoietic stem cell transplantation for DNA-double strand breakage repair disorders. J Allergy Clin Immunol. (2018) 141:322–328.e10. 10.1016/j.jaci.2017.02.036 28392333 PMC5632132

[63] Cole T McKendrick F Titman P Cant AJ Pearce MS Cale CM. Health related quality of life and emotional health in children with chronic granulomatous disease: a comparison of those managed conservatively with those that have undergone haematopoietic stem cell transplant. J Clin Immunol. (2013) 33:8–1. 10.1007/s10875-012-9758-0 23011479

